# High Level Expression and Purification of Atl, the Major Autolytic Protein of *Staphylococcus aureus*

**DOI:** 10.1155/2014/615965

**Published:** 2014-01-19

**Authors:** Vineet K. Singh

**Affiliations:** Microbiology and Immunology, Kirksville College of Osteopathic Medicine, A.T. Still University of Health Sciences, 800 West Jefferson Street, Kirksville, MO 63501, USA

## Abstract

*Staphylococcus aureus* is a major human and animal pathogen. Autolysins regulate the growth, turnover, cell lysis, biofilm formation, and the pathogenicity of *S. aureus*. Atl is the major autolysin in *S. aureus*. The biochemical and structural studies of staphylococcal Atl have been limited due to difficulty in cloning, high level overexpression, and purification of this protein. This study describes successful cloning, high level over-expression, and purification of two forms of fully functional Atl proteins. These pure proteins can be used to study the functional and structural properties of this important protein.

## 1. Introduction

*Staphylococcus aureus* is an aggressive pathogen that is responsible for a wide array of diseases from mild skin infections to life-threatening conditions such as bacteremia, pneumonia, and endocarditis [1–4]. The emergence of multidrug resistance in *S. aureus* is an enormous public health concern and there is an immediate need for additional and alternative therapeutic targets for infections caused by this bacterium [5].

Autolysins are enzymes that degrade the peptidoglycan cell wall layer and are called peptidoglycan hydrolases. They represent a diverse group of enzymes and appear to have redundant roles and more than one function [6, 7]. These enzymes include N-acetylmuramidase, N-acetyl glucosaminidase, N-acetylmuramyl-L-alanine amidase, and endopeptidase [8–11]. While amidases break the bonds between the glycan strand and the stem peptides, the glycosidases are responsible for the cleavage of the glycan strands [7].

Cellular levels and activities of autolysins are believed to be intricately regulated and are proposed to play key roles in bacterial cell wall metabolism, daughter-cell separation, and antibiotic mediated cell lysis [2, 10]. Bacteria may secrete these autolysins and cause lysis of other bacteria that compete with *S. aureus* for nutrients [12]. The peptidoglycan hydrolases are important in bacterial pathogenicity [13, 14]. These enzymes modulate muropeptide release which in turn activates host innate immune responses [13].

There is plenty of evidence to suggest that most *S. aureus* autolysins result from the processing of full-length Atl [9, 15]. The full length Atl is an ~137 kDa protein with two catalytically active domains [15–18]. A 63.3 kDa N-terminal domain possesses amidase activity while a 53.6 kDa N-terminal domain possesses the glucosaminidase activity [15–18].

Many investigators [16, 17, 19] in the past have reported difficulty in cloning the full length *S. aureus atl* gene. A difficulty in overexpression in *Escherichia coli* has also hampered functional and structural studies with this important protein. Here we report the cloning of the full-length *atl* gene in *E. coli*. Its expression was induced under appropriate conditions for large scale production of fully functional His-tagged Atl. The fully functional Atl protein was purified using a nickel affinity column and can facilitate structural and biochemical studies.

## 2. Materials and Methods

### 2.1. Bacterial Strains and Growth Conditions

*E. coli *strain JM109 (Promega) was used for all cloning experiments. In addition, *E. coli* strain BL21 (EMD Millipore) was used for all protein expression studies. Bacterial cultures were grown in Luria Bertani (LB) agar (Fisher) or in LB broth at 37°C in a static or shaking (220 rpm) incubator. When required, ampicillin (50 *μ*g mL^−1^) was added to the growth medium.

### 2.2. DNA Manipulations and Analysis

Plasmid DNA was isolated using the Qiaprep kit (Qiagen Inc.). Chromosomal DNA was isolated using a DNAZol kit (Molecular Research Center) from lysostaphin-treated *S. aureus* cells as per the manufacturer's instructions. All restriction and modification enzymes were purchased from Promega. EconoTaq DNA polymerase was purchased from Lucigen Corporation. DNA manipulations were performed using standard procedures. PCR was performed with the PTC-200 Peltier Thermal Cycler (MJ Research). Oligonucleotide primers were obtained from Eurofins MWG Operon.

### 2.3. Cloning of *S. aureus atl* Gene Fragments

**Figure 1:**
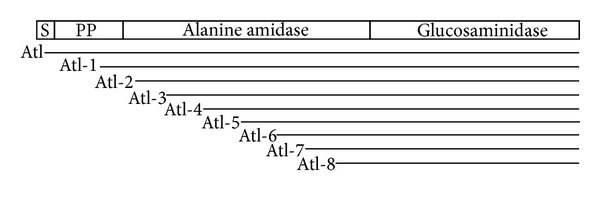
Diagrammatic representation of full-length staphylococcal *atl* and various shortened *atl* gene fragments that were cloned for overexpression in *E. coli*. The full length Atl (~137 kDa) possesses a signal peptide, a propeptide, and two catalytically active amidase and glucosaminidase domains.

The whole length *atl* gene and 8 additional *atl* gene fragments were PCR amplified using *S. aureus *RN450 [20] genomic DNA as the template. Nine forward primers and a common backward primer were used and are shown in Table 1. A *Bam*HI site (underlined) was included in the forward primers to facilitate in frame subcloning. The full-length *atl* gene amplicon included the entire coding region, the termination codon, and 10 additional downstream bases. The PCR amplicons representing the *atl* gene fragments lacked varying number of codons of the *atl*  5′-region as indicated in Table 1 in terms of the number of amino acids. These amplicons are shown diagrammatically in relation to the full length *atl* gene in Figure 1. All amplicons were cloned in the pGEMT Easy Vector System (Promega). These *atl* gene and gene fragments were subsequently subcloned in frame at the *Bam*HI and *Eco*RI sites of an overexpression vector pRSETA (Invitrogen). *E. coli* strain BL21 was subsequently transformed with the resulting nine plasmid constructs with *atl* gene fragments as described in Table 1.

### 2.4. Overexpression and Purification of Atl

The *E. coli* BL21 cells transformed with *atl* gene fragments in pRSETA were grown in LB broth containing ampicillin (50 *μ*g mL^−1^) to an OD_600_ of 0.4 and induced for the synthesis of His-tagged Atl proteins by the addition of 2.5 mM isopropyl-*β*-thiogalactopyranoside (IPTG) for 2.5 h. The induced bacterial cells were harvested and resuspended in 50 mM Tris-HCl buffer (pH 7.5) and sonicated. The sonicated extract was analyzed in 12.5% SDS-PAGE to check for over-expression of Atl.

Midexponential phase cultures of *E. coli* BL21 transformed with appropriate plasmid constructs were plated on LB agar plates with ampicillin (50 *μ*g mL^−1^) for the purification of His-tagged full length Atl and truncated Atl proteins. After overnight incubation at 37°C, the lawn of bacterial cells was recovered in 2.0 mL LB broth and added to 500 mL of fresh LB broth with ampicillin. The induced bacterial cell cultures were grown to an OD_600_ of 0.4 and induced for the production of His-tagged Atl by the addition of 2.5 mM IPTG for 2.5 h. The induced cultures were harvested and sonicated in 5 mM Tris-HCl (pH 7.9) containing 500 mM NaCl and 5 mM imidazole. The sonicated extract was centrifuged and the supernatant fluid was applied to a nickel-charged agarose affinity column (EMD Millipore) and eluted with 500 mM imidazole in 5 mM Tris-HCl (pH 7.9) containing 500 mM NaCl after appropriate washes as recommended by the manufacturer. Fractions containing the overexpressed His-tagged Atl were pooled, dialyzed, and concentrated against 50 mM Tri-HCl, pH 7.2. The dialyzed Atl proteins were assessed for purity and used for the detection of autolytic activity.

### 2.5. Determination of Lytic Activity of Atl Proteins

Lytic activities of different His-tagged Atl proteins were detected using a zymographic procedure as described previously [21, 22] using autoclaved *S. aureus* RN450 cells impregnated in 12.5% SDS-PAGE gels. The gels after electrophoresis were renatured for the autolysins by incubation in 50 mM Tri-HCl buffer, pH 7.2, containing 0.05% Triton X-100.

Lytic activities of purified His-tagged Atl proteins were also determined by measuring the loss of turbidity of a staphylococcal cell suspension. Autoclaved *S. aureus* RN450 cells were suspended in 50 mM Tri-HCl buffer, pH 7.2, containing 0.05% Triton X-100 to an OD_600_ = 1.0. Purified Atl proteins (50 *μ*g) were added to 1.0 mL of the suspension and the decrease in turbidity (OD_600_) after 1 h was measured spectrophotometrically.

## 3. Results and Discussion

### 3.1. Cloning, Overexpression, and Purification of Atl

Subsequent to PCR amplification, we successfully cloned and then sub-cloned the full length *atl *gene and eight truncated *atl* genes from the 5′-end in the vector pRSETA (Table 1). All these DNA fragments were downstream and in frame in pRSETA with a sequence that encoded an N-terminal fusion peptide of 34 amino acids. This peptide contained a polyhistidine tag as a metal binding domain that facilitated purification of the overexpressed recombinant Atl protein on a nickel affinity column.

**Figure 2:**
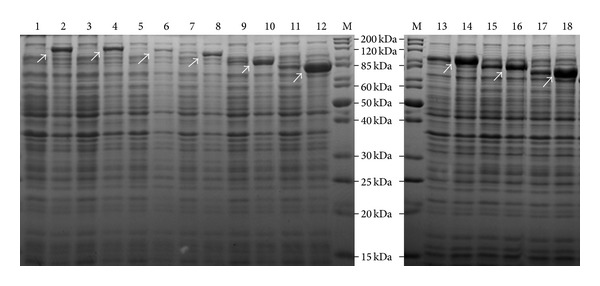
Coomassie stained gels demonstrating overexpression of recombinant Atl. The 12.5% SDS-PAGE contains protein extracts of *E. coli* BL21 cells with pRSETA plasmids expressing either full length or truncated Atl proteins. The odd number labels are cells grown without IPTG and the even number labels are the cells grown with IPTG. Lane M: standard protein markers; lanes 1 and 2 - Atl (full length His-tagged Atl); lanes 3 and 4: Atl-1; lanes 5 and 6: Atl-2; lanes 7 and 8: Atl-3; lanes 9 and 10: Atl-4; lanes 11 and 12: Atl-6; lanes 13 and 14: Atl-5; lanes 15 and 16: Atl-7; lanes 17 and 18: Atl-8. The Atl number suffixes are indicated in Table 1.

*E. coli* strain BL21 was transformed with pRSETA plasmid containing *atl* gene or various *atl* gene fragments as shown in Table 1. The recombinant His-tagged Atl proteins were successfully overexpressed by the addition of IPTG to the mid-exponential phase cultures of appropriate transformants (Figure 2). It is evident that the full-length as well as all truncated recombinant Atl proteins with the exception of Atl-2 (lane 6, Figure 2) were produced at a very high level in IPTG treated bacterial cells (short arrows, Figure 2). The apparent size of the overexpressed recombinant His-tagged Atl proteins matched closely with the predicted molecular weight of these proteins (Table 1 and Figure 2).

**Figure 3:**
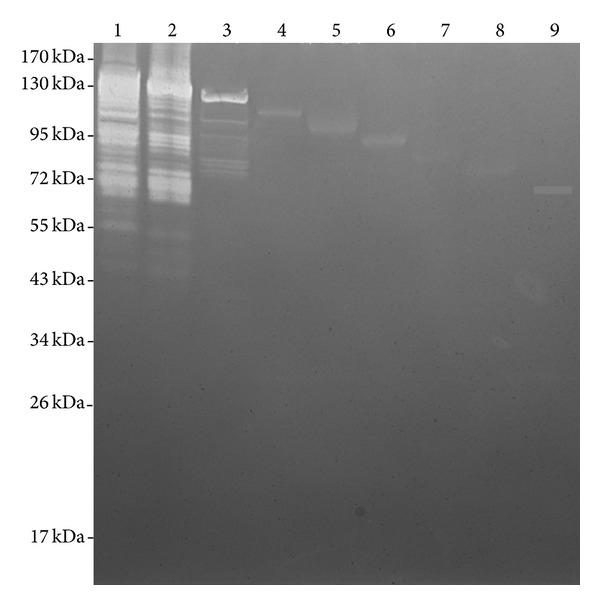
Zymographic detection of the autolytic activity in recombinant His-tagged Atl. Protein extracts from *E. coli* BL21 cells grown with IPTG were used in this study. The protein extract was separated by 12.5% SDS-gel electrophoresis. The SDS gel was impregnated with autoclaved *S. aureus* RN450 cells. Autolytic activity was detected by renaturing the autolysins by incubation of the gel in 50 mM Tri-HCl buffer, pH 7.5, containing 0.05% Triton X-100. Lane 1: full length His-tagged Atl; lane 2: Atl-1; lane 3: Atl-2; lane 4: Atl-3; lane 5: Atl-4; lane 6: Atl-5; lane 7: Atl-6; lane 8: Atl-7; lane 9: Atl-8.

A zymographic technique was used to assess the autolytic activity of the recombinant Atl proteins. Autoclaved *S. aureus* RN450 cells were impregnated in the 12.5% SDS gel that was used to separate the *E. coli *cell extract containing the recombinant Atl proteins. When these SDS gels were renatured, the lanes representing the *E. coli *extract with full length N-terminal and C-terminal catalytic domains of Atl (Figure 3, Atl and Atl-1 in lanes 1 and 2, resp.) showed the highest activity with multiple lytic bands. The recombinant Atl proteins that possess only the C-terminal glucosaminidase activity domain but truncated amidase domain did not result in any visible autolytic activity in the zymography (Figure 3, lanes 4–9). Truncated Atl proteins were produced at a very high level as can be seen in Figure 2 (lanes 8–18). In the zymography, the presence of these highly overexpressed proteins can be seen as a single dense band. There was no autolytic activity associated with these protein bands. Although Atl-2, with slight deletion of the N-terminal region of the amidase domain, demonstrated catalytic activity in the zymogram (Figure 3, lane 6), its expression level was low in *E. coli* BL21 cells under identical conditions that mimicked very high level expression of all other recombinant Atl proteins (Figure 2). This low level of Atl-2 may account for the limited lytic activity in the corresponding lane in the zymography (Figure 3, lane 3).

The purified recombinant His-tagged Atl proteins were also used to determine the lytic activity against staphylococcal cells. Consistent with the zymographic data, pronounced lytic activity was seen only with full length Atl and Atl-1 and slight activity with Atl-2 (Table 2). No lytic activity was observed with any of the other recombinant His-tagged Atl proteins with larger deletions of the amidase domain (Table 2).

**Figure 4:**
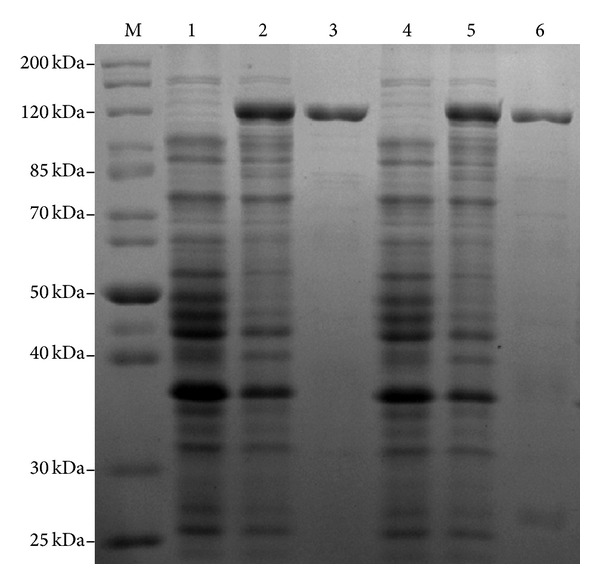
Coomassie stained gels showing purity of recombinant His-tagged Atl. Recombinant Atl and Atl-1 proteins were overproduced in *E. coli* and purified as described in the Materials and Methods section. Lane M: standard protein markers; lanes 1 and 2: protein extract from *E. coli* BL21 cells transformed with plasmids pRSETA-*atl* and grown without and with IPTG, respectively; lane 3: purified His-tagged Atl; lanes 4 and 5: protein extract from *E. coli* BL21 cells transformed with plasmids pRSETA-*atl1* and grown without and with IPTG, respectively; lane 6: purified His-tagged Atl-1.

**Figure 5:**
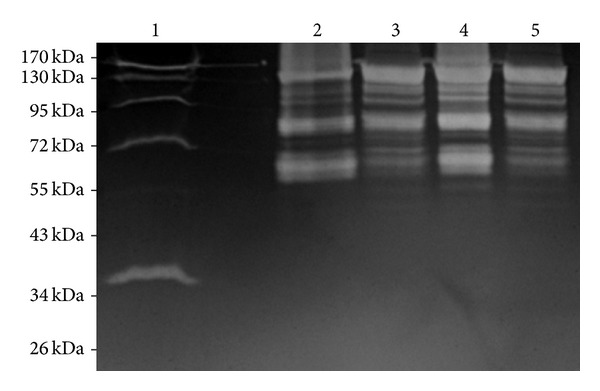
Zymographic detection of the autolytic activity in purified recombinant His-tagged Atl. Lane 1: total protein extract from *S. aureus* cells. Lanes 2 and 4: purified His-tagged Atl and Atl-1, respectively; lanes 3 and 5: protein extract from *E. coli* BL21 cells transformed with plasmids pRSETA-*atl* or pRSETA-*atl1 *and grown with IPTG, respectively.

During overexpression of the recombinant His-tagged Atl proteins, IPTG was added to the *E. coli* BL21 cultures with appropriate plasmids at an OD_600_ = 0.4. In 2.5 h after the addition of IPTG, the final OD_600_ of these cultures was around 0.65. We have used *E. coli* cells transformed with pRSETA with many cloned genes to overexpress various staphylococcal proteins for their characterization [11, 23–26] and, based on our previous experiences, we did not see any toxic effect of recombinant Atl on the *E. coli* BL21 cells. Using a nickel-charged agarose affinity column, we were able to successfully purify up to 4.0 mg of recombinant Atl or Atl-1 from a 500 mL IPTG-treated culture. Subsequent to nickel affinity column chromatography, the purity of recombinant Atl and Atl-1 was assessed by 12.5% SDS-PAGE. A single step column purification yielded reasonably pure forms of recombinant Atl and Atl-1 (Figure 4, lanes 3 & 6, resp.). The autolytic activities of these pure recombinant Atl and Atl-1 proteins were assessed using the zymographic technique. In zymographic analysis, the autolytic activity bands of recombinant Atl and Atl-1 ranged from 141 kDa to 43 kDa (Figures 3 and 5). The lytic bands resulting from recombinant Atl and Atl-1 can also be seen with respect to the lytic bands resulting from the total protein extract from *S. aureus* cells (Figure 3, lanes 1–5). Presence of multiple lytic bands associated with even pure Atl and Atl-1 supports the view that most *S. aureus* autolysins result from the processing of full length Atl [9, 15]. The autolytic pattern of the purified recombinant Atl and Atl-1 that is similar to the crude *E. coli* extract containing these proteins (Figure 5, lanes 2 & 3, lanes 4 & 5) suggests that the processing of staphylococcal Atl is to a large extent an inherent process.

Many investigators have experienced problems in cloning and over-expression of full length staphylococcal Atl [16, 17, 19]. However, Hirschhausen et al. (2010) recently expressed full length Atl and different Atl-subdomains in *E. coli *[27]. Using these recombinant proteins, they were able to demonstrate that Atl mediates internalization of *S. aureus* cells by the nonprofessional phagocytic cells [27]. However, it is unclear what level of overexpression was obtained by Hirschhausen et al. [27].

Many roles have been proposed and shown experimentally for the staphylococcal Atl. It is postulated that Atl plays role in cell division [9, 15–17]. By generating enzymatically inactive point mutations, it has been shown that both the amidase and the glucosaminidase domains of Atl must be catalytically active for *S. aureus* to form a biofilm [16]. Consistent with this finding, it has been shown that gallidermin, prevents biofilm formation by *S. aureus*. This lantibiotic also repressed the transcription of the gene encoding Atl [28]. Staphylococcal Atl is also reported to be involved in the excretion of cytoplasmic proteins. In a comparative analysis, many of the cytoplasmic proteins that are excreted by wild-type *S. aureus* cells, were significantly decreased in an *atl* mutant [29]. It has also been shown that *atl* mutants are significantly reduced in their capacities to be internalized by endothelial cells [27]. Atl mediated internalization of *S. aureus* cells by non-professional phagocytes may assist staphylococci in evading the host immune system [27]. Additionally, an autolysis-defective mutant showed reduced virulence in a rat endocarditis model, suggesting that autolysins are important for *S. aureus* pathogenicity [30].

In view of the ability of the autolysins to target the bacterial cell wall and regulate cell wall associated processes, the autolysins are being considered as effective antimicrobials with potentially important applications in medicine and biotechnology [31]. In addition, autolysins are also being targeted as potential vaccine candidates [32]. A detailed and insightful study with staphylococcal Atl has been limited thus far by the lack of the ability to make this protein in functional form and in large quantity. The plasmid constructs and the *E. coli* strains described in this study could be used to produce large amounts of functional Atl proteins that can be purified by one-step affinity chromatography. The purified Atl could then be used for more biochemical and structural studies and may lead to strategies to control infections caused by *S. aureus*.

## Figures and Tables

**Table 1:** Oligonucleotide primers used in this study.

Primer	Sequence	Amplicon^1^	Plasmid^2^	Protein ID	Predicted MW/PI^3^	Number of amino acids absent^4^
Atl-FW	GGATCCATGGCGAAAAAATTCAATTAC	3790	pRSETA-*atl *	Atl	141258/9.59	0
Atl-FW-1	GGATCCGCTGGTTATAGTTTAGTTGATG	3382	pRSETA-*atl1 *	Atl-1	126872/9.57	136
Atl-FW-2	GGATCCCCACAAGTAAACTCTTCAAT	3136	pRSETA-*atl2 *	Atl-2	118310/9.59	218
Atl-FW-3	GGATCCCATGCATTTGTTGATGGGGATCG	2920	pRSETA-*atl3 *	Atl-3	110038/9.62	290
Atl-FW-4	GGATCCGGTGGTACTGACCATGCCGATCC	2659	pRSETA-*atl4 *	Atl-4	100288/9.73	377
Atl-FW-5	GGATCCGTATACGACAAAACTGGTAAAG	2401	pRSETA-*atl5 *	Atl-5	91053/9.75	463
Atl-FW-6	GGATCCGTGTCTGGCTCTGGAAACCAAACA	2143	pRSETA-*atl6 *	Atl-6	81641/9.75	549
Atl-FW-7	GGATCCAACGGTGTTGCTCAAATTAATGC	1942	pRSETA-*atl7 *	Atl-7	74557/9.71	616
Atl-FW-8	GGATCCTCACCTGTAAATGTAATGCAAAC	1729	pRSETA-*atl8 *	Atl-8	66778/9.71	687
Atl-RV	GAATTCATGTTGCTTATTTATATTG					

All PCR were carried out using the indicated forward primers and a common reverse primer. The forward primers included a *Bam*HI site (underlined) and the reverse primer included an *Eco*RI site (underlined) to facilitate the subcloning of the amplified fragment. ^1^The size of the resulting amplicon is indicated in base pairs. ^2^The construct where the corresponding amplicon was cloned in vector pRSETA at *Bam*HI and *Eco*RI sites. ^3^The molecular weight (MW) and the PI of the recombinant proteins likely to be expressed in *E. coli*  BL21 with the corresponding plasmid constructs. The MW/PI was calculated using the website http://web.expasy.org/compute_pi/. ^4^The numbers indicate the lack of Atl N-terminal amino acids. All recombinant Atl proteins include an extra 34 amino acids at the N-terminus that arises from the vector pRSETA.

**Table 2:** Lytic activity of purified recombinant His-tagged Atl proteins.

Protein	% decrease in turbidity*
Atl	56.76 ± 2.09
Atl-1	50.69 ± 5.50
Atl-2	5.73 ± 0.32
Atl-3	No decrease
Atl-4	No decrease
Atl-5	No decrease
Atl-6	No decrease
Atl-7	No decrease
Atl-8	No decrease

*Average of three independent experiments ± standard deviation.
